# Supplementary material for: A standardized metric to enhance clinical trial design and outcome interpretation in type 1 diabetes
Source: Nat Commun. 2023 Nov 8;14:7214. doi: 10.1038/s41467-023-42581-z (PMC10632453; doi:10.1038/s41467-023-42581-z)
Supplement: Supplementary file 1 — Supplementary Information [file 41467_2023_42581_MOESM1_ESM.pdf]

A Standardized Metric to Enhance Clinical Trial Design and Outcome Interpretation in Type 1 Diabetes

Alyssa Ylescupidez[1†], Henry T. Bahnson[1†], Colin O'Rourke[1], Sandra Lord[1], Cate Speake[1‡], Carla J. Greenbaum[1‡]

[1]Center for Interventional Immunology, Benaroya Research Institute at Virginia Mason; Seattle, WA, USA.

[†]Authors contributed equally.

‡Authors contributed equally.

Supplementary Figures:

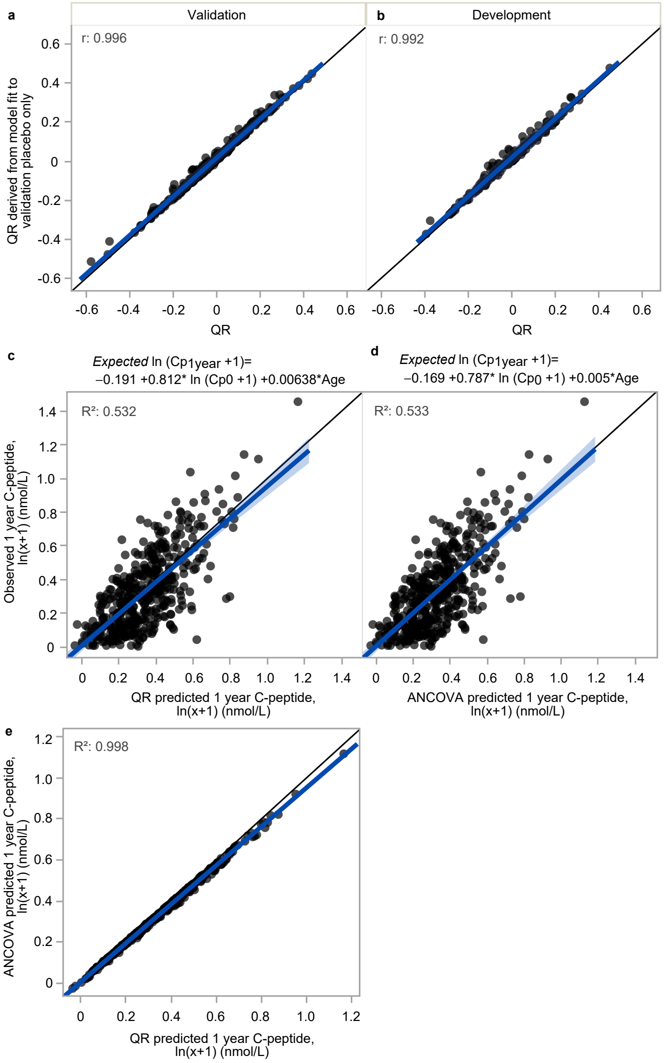

Supplemental Figure 1. No difference between original and revised ANCOVA model to determine QR.

QR derived from ANCOVA fit to validation cohort placebo/control participants only is strongly correlated with published QR model (i.e., model derived from development cohort) within both (**a**) validation (n=286) and (**b**) development (n=162) cohorts. Original ANCOVA model from 5 studies used in development of original QR model (**c**) compared with revised ANCOVA model using placebo/control participants from all 13 studies, n=448 (**d**). Predictive values between two models are strongly associated (R^2 =0.998) (**e**). Blue line indicates linear regression lines and shaded blue regions are 95% confidence intervals.

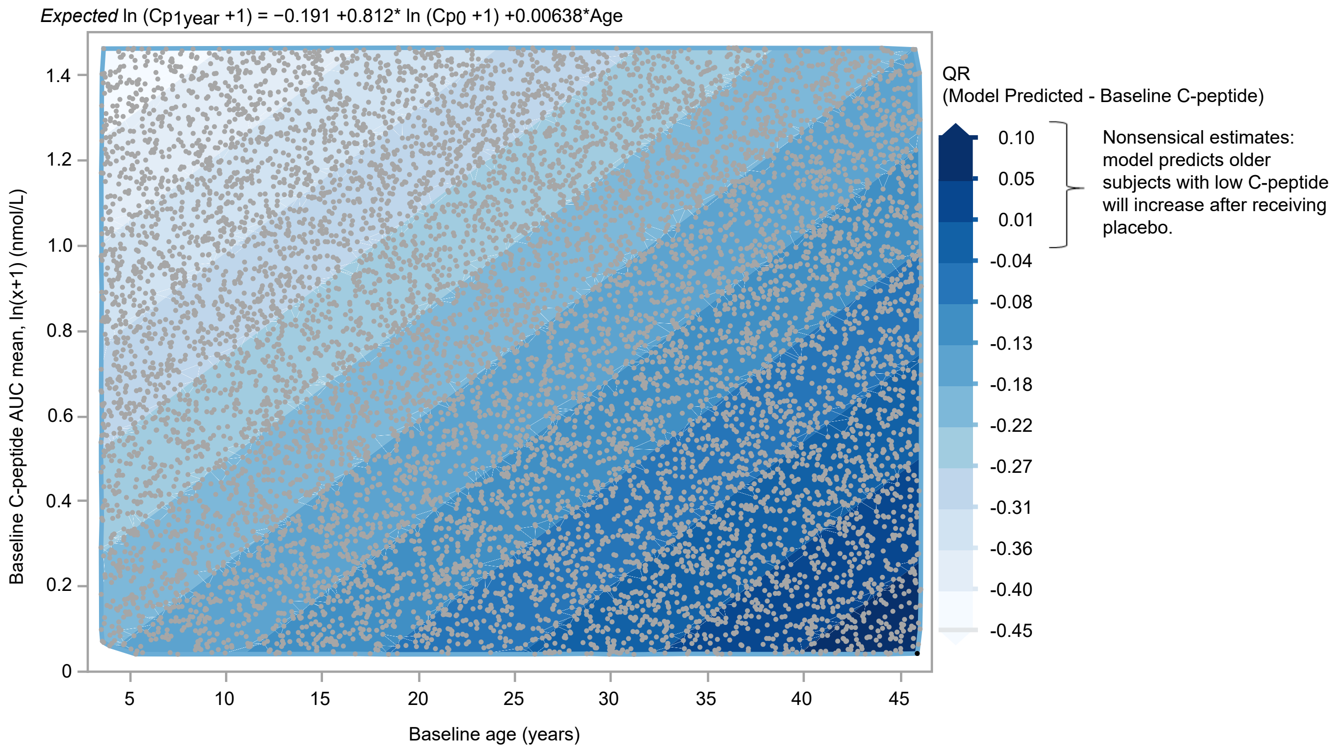

Supplemental Figure 2. Evaluation of model predicted C-peptide across wide range of baseline C-peptide values and ages.

Contour plot of model predicted C-peptide subtracted from baseline C-peptide across wide range of ages and baseline C-peptide. Dark blue regions in the lower right quadrant of the figure correspond to older subjects with low levels of baseline C-peptide where the model predicts subjects to have no decline or minor positive increase in C-peptide at 1 year.

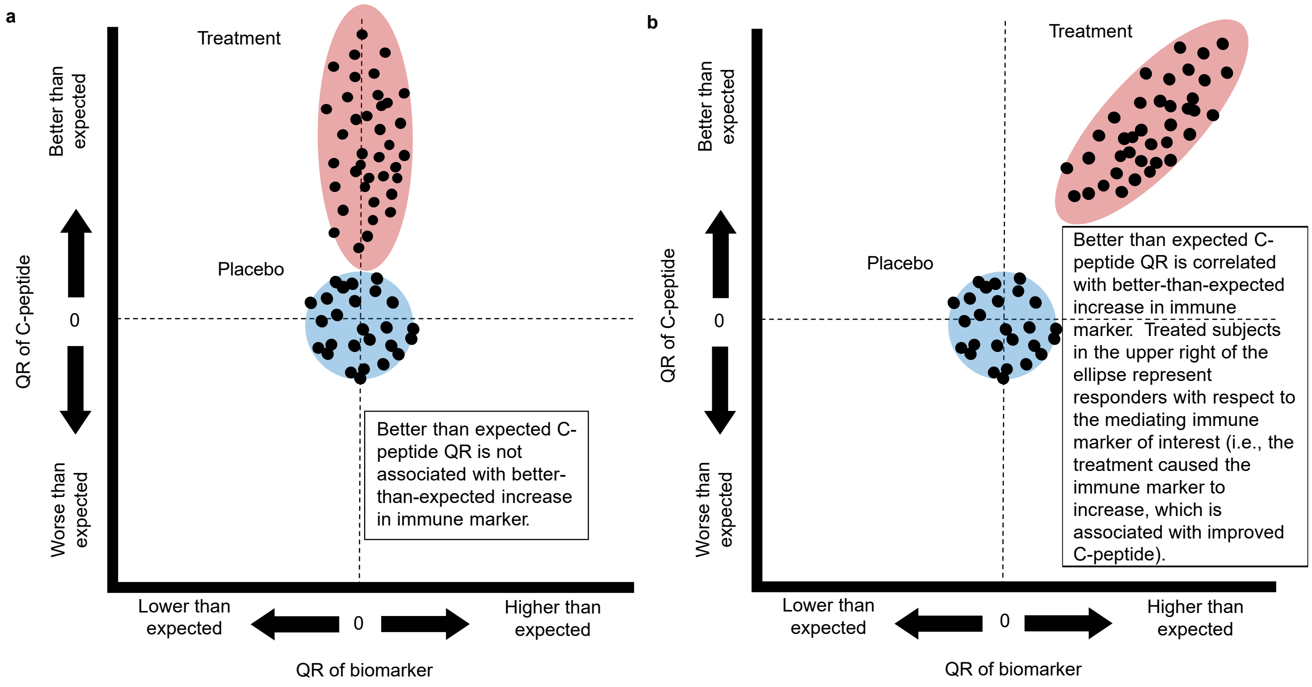

Supplemental Figure 3. Multivariable adjustment with QR allows for assessment of causal association between biomarker and C-peptide.

(**a**) Treatment is effective, but not associated with immune marker. (**b**) Evidence of causal association: better C-peptide response is correlated with biomarker among treated individuals.

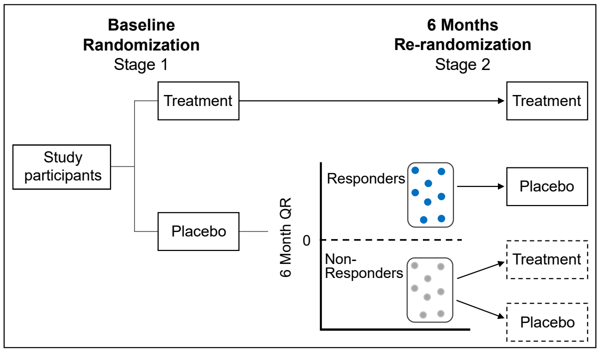

Supplemental Figure 4. Sequential parallel comparison design (SPCD) to improve trial efficiency.

SPCD re-randomizes placebo non-responders at 6 months and provides opportunity for additional individuals to potentially benefit from treatment as well as increasing efficiency of trial.
