# Supplementary material for: A standardized metric to enhance clinical trial design and outcome interpretation in type 1 diabetes
Source: Nat Commun. 2023 Nov 8;14:7214. doi: 10.1038/s41467-023-42581-z (PMC10632453; doi:10.1038/s41467-023-42581-z)
Supplement: Supplementary file 3 — Reporting Summary [file 41467_2023_42581_MOESM3_ESM.pdf]

nature portfolio

Corresponding author(s): C. Speake and C.J. Greenbaum

Last updated by author(s): 2023/08/29

Reporting Summary

Nature Portfolio wishes to improve the reproducibility of the work that we publish. This form provides structure for consistency and transparency in reporting. For further information on Nature Portfolio policies, see our Editorial Policies and the Editorial Policy Checklist.

Statistics

For all statistical analyses, confirm that the following items are present in the figure legend, table legend, main text, or Methods section.

n/a	Confirmed	
☐	☒	The exact sample size (*n*) for each experimental group/condition, given as a discrete number and unit of measurement
☒	☐	A statement on whether measurements were taken from distinct samples or whether the same sample was measured repeatedly
☐	☒	The statistical test(s) used AND whether they are one- or two-sided<br>*Only common tests should be described solely by name; describe more complex techniques in the Methods section.*
☐	☒	A description of all covariates tested
☐	☒	A description of any assumptions or corrections, such as tests of normality and adjustment for multiple comparisons
☐	☒	A full description of the statistical parameters including central tendency (e.g. means) or other basic estimates (e.g. regression coefficient) AND variation (e.g. standard deviation) or associated estimates of uncertainty (e.g. confidence intervals)
☐	☒	For null hypothesis testing, the test statistic (e.g. *F*, *t*, *r*) with confidence intervals, effect sizes, degrees of freedom and *P* value noted<br>*Give P values as exact values whenever suitable.*
☒	☐	For Bayesian analysis, information on the choice of priors and Markov chain Monte Carlo settings
☒	☐	For hierarchical and complex designs, identification of the appropriate level for tests and full reporting of outcomes
☐	☒	Estimates of effect sizes (e.g. Cohen's *d*, Pearson's *r*), indicating how they were calculated

Our web collection on statistics for biologists contains articles on many of the points above.

Software and code

Policy information about availability of computer code

Data collection	No data collection was involved in this study; all data obtained are from previously published clinical trials.
Data analysis	Data analyses were conducted using JMP Pro 16 (SAS Institute Inc., Cary, NC, USA) and SAS 9.4 (SAS Institute Inc., Cary, NC, USA).

For manuscripts utilizing custom algorithms or software that are central to the research but not yet described in published literature, software must be made available to editors and reviewers. We strongly encourage code deposition in a community repository (e.g. GitHub). See the Nature Portfolio guidelines for submitting code & software for further information.

Data

Policy information about availability of data

All manuscripts must include a data availability statement. This statement should provide the following information, where applicable:

- Accession codes, unique identifiers, or web links for publicly available datasets
- A description of any restrictions on data availability
- For clinical datasets or third party data, please ensure that the statement adheres to our policy

Clinical trial data analyzed in this study are available under restricted access due to data privacy laws. TrialNet clinical trials data can be obtained by application to the NIDDK central repository at https://repository.niddk.nih.gov/home/. Immune Tolerance Network clinical trials data is freely available at https://www.itntrialshare.org/.

Human research participants

Policy information about studies involving human research participants and Sex and Gender in Research.

Reporting on sex and gender	The incidence of type 1 diabetes does not vary by sex or gender. Here, we apply, validate, and extend a model to predict T1D progression. The original developers of the model found that sex "contributed nothing to reducing the unexplained variance, p=0.941" of their predictive model (Bundy et al 2016). For these reasons, we did not include sex in our validation and extension of the model.
Population characteristics	Please see Table 1 of our manuscript.
Recruitment	No individuals were specifically recruited for this analysis. We utilized data from 13 clinical trials enrolling new onset type 1 diabetes subjects. These 13 trials occurred between 2004 and 2020, included individuals from 3 to 46 years of age, were conducted at multiple locations, and included data from both academic trials and a phase 3 industry-sponsored study.
Ethics oversight	We are utilizing existing data from previously published trials all of which were conducted with IRB/ethics board oversight and appropriate consent and assent from participants as described in the original study reports. Clinical trial registration numbers are provided in the manuscript for all studies, so that full information regarding each can be gleaned.

Note that full information on the approval of the study protocol must also be provided in the manuscript.

Field-specific reporting

Please select the one below that is the best fit for your research. If you are not sure, read the appropriate sections before making your selection.

[x] Life sciences [ ] Behavioural & social sciences [ ] Ecological, evolutionary & environmental sciences

For a reference copy of the document with all sections, see nature.com/documents/nr-reporting-summary-flat.pdf

Life sciences study design

All studies must disclose on these points even when the disclosure is negative.

Sample size	We are utilizing existing data from all participants in previously published trials. Information about sample sizes and power from the original clinical trials is available in the original study reports.
Data exclusions	Data were only excluded due to missingness; a complete case analysis was performed, where individuals were excluded only if QR could not be computed due to missing C-peptide data at the timepoint of interest.
Replication	We successfully demonstrate replication of the original published model (see figure 1).
Randomization	N/A. We utilized deidentified data from previously published trials all of which were randomized. The specific details for each trial is available in the original publications of the trial results..
Blinding	N/A. We utilized deidentified data from these previously published trials. The specific details for each trial is available in the original publications of the trial results..

Reporting for specific materials, systems and methods

We require information from authors about some types of materials, experimental systems and methods used in many studies. Here, indicate whether each material, system or method listed is relevant to your study. If you are not sure if a list item applies to your research, read the appropriate section before selecting a response.

Materials & experimental systems

n/a	Involved in the study	
[x]	[ ]	Antibodies
[x]	[ ]	Eukaryotic cell lines
[x]	[ ]	Palaeontology and archaeology
[x]	[ ]	Animals and other organisms
[ ]	[x]	Clinical data
[x]	[ ]	Dual use research of concern

Methods

n/a	Involved in the study	
[x]	[ ]	ChIP-seq
[x]	[ ]	Flow cytometry
[x]	[ ]	MRI-based neuroimaging

Clinical data

Policy information about clinical studies

All manuscripts should comply with the ICMJEguidelines for publication of clinical research and a completedCONSORT checklist must be included with all submissions.

Clinical trial registration	De-identified data were obtained from 13 clinical trials of individuals with recently diagnosed T1D (Table 1 of manuscript). The clinical trial registration numbers are: NCT00100178, NCT00279305, NCT00529399, NCT00505375, NCT009474276, NCT02215200, NCT02067923, NCT00965458, NCT00515099, NCT02293837, NCT01781975, NCT00435981, NCT00723411.
Study protocol	Full trial protocols are available alongside the initial trial publications, at the ITN Trialshare website (https://www.immunetolerance.org/researchers/trialshare), or from the authors of this manuscript.
Data collection	These 13 trials occurred between 2004 and 2020, included individuals from 3 to 46 years of age, were conducted in the US, Canada, and Europe, and included data from both academic trials and a phase 3 industry-sponsored study. For a complete listing of sites and details of the original trials, original trial reports are referenced in the manuscript, and available at clinicaltrials.gov via the registration numbers noted above and in the manuscript. No new data were generated in the current study.
Outcomes	N/A; we are not reporting results of a clinical trial, instead are using data from existing previously published studies.
